# Concentric Circle Technique for Assessment of Femoral Head Deficiency in Osteonecrosis of Femoral Head

**DOI:** 10.7759/cureus.18285

**Published:** 2021-09-25

**Authors:** Arvind Kumar, Siddhartha Sinha, Javed Jameel, Sandeep Kumar

**Affiliations:** 1 Orthopaedics, Hamdard Institute of Medical Sciences and Research, New Delhi, IND

**Keywords:** radiographic assessment, osteonecrosis, femoral head, collapse, avn

## Abstract

Introduction

There is a lack of standardized objective tools to assess collapse for osteonecrosis of the femoral head (ONFH) patients’ follow-up. We describe a quantitative technique of collapse assessment using a superimposed concentric circular ring matching the intact part of the femoral head in anteroposterior (AP) radiographs.

Methods

We retrospectively analyzed 30 normal femoral heads and 30 ONFH (15 pre-collapse,15 post-collapse) in anteroposterior hip radiographs. A best-fitting circle was superimposed on articular margins of the femoral head and the maximum width of the deficient zone of the femoral head (not matching the circle) was measured. The width (pW) was measured as percentage-width in proportion to the circle’s diameter. The findings were compared among normal and ONFH radiographs. Intraclass correlation coefficients were calculated for intraobserver and interobserver reliability of the measurements.

Results

The mean femoral head deficiencies predicted by pW were 0.2±0.5% for normal hip, 2.8±1.1% for pre-collapse, and 8.9±3.8% for post-collapse radiographs. We observed significant differences in the measurements of pW among the control group, pre-collapse and post-collapse groups. Interobserver and intraobserver reliabilities for the measurements were high.

Conclusion

The described concentric circle technique is a simple and reliable method for objective assessment of subtle alterations in the sphericity of the femoral head and can be helpful for the radiographic follow-up of ONFH patients.

## Introduction

Osteonecrosis of the femoral head (ONFH) is a debilitating hip joint condition, usually affecting the young population [1]. Core decompression procedures alone or with bio-supplementation using agents like platelet-rich plasma, mesenchymal stem cells, and synthetic bone grafts have satisfactory outcomes in early ONFH [2,3]. However, these procedures do not warrant a complete cure of the disease, and the osteonecrosis or secondary joint changes may persist and progress [4]. In addition, the morphological alteration in the shape of the femoral head, especially the weight-bearing zone, has a prognostic role in the long-term consequences of ONFH [5]. The widely used modified Ficat and Arlet classification classified the ONFH into five stages. The first three stages (0-IIa) are pre-collapse stages, and the last two (III and IV) are post-collapse stages [6]. Stage IIb is a transition stage when the subchondral collapse commences and progresses to stage III (flattening). The outcomes of hip preservation procedures are poor once the collapse ensues because the sphericity of the femoral head, which is an integral component of femoroacetabular articulation, is lost [7]. Therefore, all efforts should be made to halt the progression of pre-collapse stages towards the post-collapse stages. The advanced core decompression measures that use bone graft and bone graft substitute effectively manage voids resulting from the debridement of osteonecrosis cavities. These techniques are best utilized in pre-collapse stages [8]. Considering the risk of collapse in ONFH, timely detection of even minor changes in the femoral sphericity can help prevent collapse by bone graft or bone graft substitute-based augmentation, as in advanced core decompression. In addition, for patients who already underwent the core decompression procedures, a change in sphericity of the femoral head will have a prognostic role [9]. However, the clinical follow-up in ONFH patients is usually supplemented by follow-up radiographs for brief intervals and magnetic resonance imaging (MRI) for longer intervals [10]. During follow-up, the radiographic observations are mainly subjective, and minor femoral head shape changes are bound to be missed.

Standard anteroposterior (AP) radiographs can be easily performed in ONFH patients. However, the lateral radiographs may be difficult to standardize due to difficult limb positioning because of limited rotations and abduction in many patients. Therefore, we devised a simple quantitative method to assess collapse in AP radiographs of the hip joint objectively. In our technique of collapse assessment, we superimposed a concentric circular ring matching the intact part of the femoral head in AP radiographs. Then, we measured the maximum width of the deficient part of the circle in the collapsed zone of the femoral head. The current study aims at analyzing the applicability of the aforestated technique as an objective tool for radiographic assessment of collapse in ONFH.

## Materials and methods

We retrospectively collected 30 AP radiographs of normal hip joints (control group) and 30 AP radiographs of ONFH affected hip joints (diseased group) for analysis. The radiographs were stored as digital copies in the hospital database and were collected in an identity-anonymized form. In both groups, we included the radiographs belonging to patients aged 18 to 40 years, and radiographs with gross abnormalities, except for the ONFH-related findings in the diseased group, were excluded. These abnormalities included evidence of joint degeneration, deformities, congenital anomalies, bone tumors, irregular bones, and those with signs of old fractures or surgery. In addition, half of the radiographs in the diseased group were kept from pre-collapse stages (up to Modified Ficat Arlet stage IIA) and another half from the post-collapse stages (Modified Ficat Arlet stages IIb and III, excluding stage IV) based upon the documented diagnosis.

**Figure 1:**
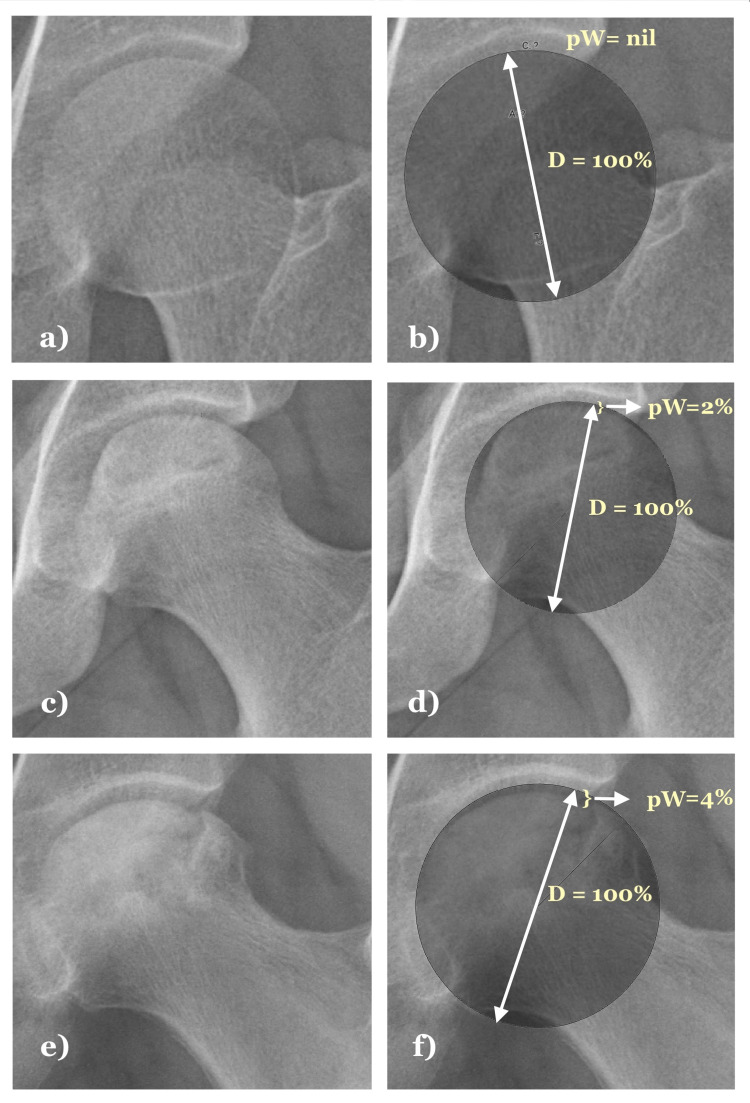
Assessment of femoral head deficiency by superimposing a matching diameter circle in hip AP radiographs. Nil deficiency is seen in a normal femoral head (a, b). Subtle deficiency observed in pre-collapse ONFH affected hip radiograph (c, d). Larger deficiency observed in post-collapse ONFH affected hip radiograph (e, f). ONFH: Osteonecrosis of the femoral head

The technique of assessment of femoral head collapse (Figure 1):

1) The digital copies of the hip AP radiographs were assessed. A best-fitting circle was superimposed over the femoral head articular extent by closely matching its medial, inferomedial, and lateral-most articular contour.

2) The deficient segment of the femoral head lying beneath the circle’s extent was located. After that, the widest extent of the deficient zone of the femoral head is marked on the circle.

3) A straight line is extended from the marked point towards the circle’s center and further to the opposite circular margin. The line corresponds to the circle’s diameter, which also represents the diameter of the femoral head. The deficient zone’s width was measured as the percentage of the circle’s diameter. Thus, the percentage width of the deficient zone (pW) = (measured width/measured diameter) x 100.

4) The measurement of the "pW" helps avoid errors of absolute metric measurements due to variable magnification. This percentage measurement also standardizes collapse assessment for each radiograph without getting affected by the individual variations of femoral head size. We used Imagemeter ™ software for drawing the best fitting circles and other measurements.

The measurements were separately made by two trainee residents, two senior residents, and two faculties in orthopedics. The radiographs' groups were not disclosed to the measuring observers. The means of their findings for individual radiographs were considered as the final "pW" values. Intraclass correlation coefficients (ICC) were calculated for intraobserver and interobserver reliability of the measurements made by the six participants. The coefficient values below 0.50 were grade as poor, between 0.50 and 0.75 as moderate, between 0.75-0.90 as good, and above 0.90 as excellent [11]. Finally, we calculated the mean±standard deviation and range of the final "pW" values among the control group and the subgroups of the diseased group. The findings among the groups were compared using one-way ANOVA (Analysis of variance) and pairwise comparisons for significant differences. A p-value of less than 0.05 was considered statistically significant.

## Results

The mean femoral head deficiencies measured by "pW" were 0.2±0.5% for normal hip radiographs, 2.8±1.1% for precollapse radiographs, and 8.9±3.8% for the post-collapse radiographs. The ICC for intraobserver reliability of the measurements was 0.99 for all participants. The ICC for the interobserver reliability among the six observers was 0.98. We observed significant differences in the measurement of "pW" among the control group, pre-collapse and post-collapse groups. The deficient zone was nil in 83.33% (25 out of 30) normal hip radiographs.

Interestingly, all 15 radiographs marked precollapse in prior radiological assessment had evidence of deficient femoral heads that could not match a perfect circle. However, the deficient zone in the precollapse stage was significantly low compared to the post-collapse ONFH radiographs. The detailed results are provided in Table 1.

**Table 1:** Comparison of femoral head deficiency in normal, pre-collapse, and post-collapse ONFH radiographs. ^Pair 1- Control group: Pre-collapse group; Pair 2- Control group: Post-collapse group; Pair 3- Pre-collapse group: Post-collapse group ONFH: Osteonecrosis of the femoral head

Parameters	Control group (n = 30)	Pre-collapse ONFH (n = 15)	Post collapse ONFH (n = 15)	Statistical significance
Deficient zone of femoral head, pW (Mean±SD)	0.2±0.5%	2.8±1.1%	8.9±3.8%	Significant differences of pW among the three radiographic groups, p < 0.05 (ANOVA) Pairwise comparisons: Significant differences among the three pairs, p < 0.05^
Range	0-1.63%	1.43-5.46%	4.35-15.63%	-
Nil deficient zone/pW=0 (number of cases, (%))	25 (83.33%)	0 (0%)	0 (0%)	-

## Discussion

The current study describes a simple technique for collapse assessment in ONFH. As discussed earlier, the collapse and its progression are associated with poor outcomes due to the loss of the femoral head’s sphericity. Therefore, timely detection of even minor changes in the femoral head can help in individualized care for ONFH patients. The care can be surgical through bone graft augmentation (advanced core decompression) or activity modification until a radiological consolidation of the ONFH lesion is evident. Our findings suggest that a small proportion of normal hip joints (16.66%) can also have a minor deviation from the round circular contour. However, the deviation is clinically irrelevant since the actual conversion of "pW" into a metric value will correspond to a sub-millimeter measurement.

On the other hand, all radiographs of previously diagnosed precollapse stages of ONFH had subtle deficiencies in the femoral head that might have got missed in subjective assessment of radiographs. The importance of monitoring the deficient zone lies in predicting an impending collapse. It means that a non-progressive deficiency of the femoral head suggests a stable osteonecrotic lesion. In contrast, a progressive increase in the deficiency can potentially result in an acute collapse of the necrotic zone. The post-collapse ONFH radiographs are bound to have a major deficiency of the femoral head. Therefore, our findings suggest a significant difference in the measurement of "pW" among the precollapse and post-collapse groups.

Furthermore, the described technique has good interobserver and intraobserver correlation. Therefore, the described technique can act as an objective tool for measuring and monitoring collapse in ONFH patients. The technique is simple and involves superimposing a circle over the AP radiographic projection of the femoral head. Since the circle’s diameter is its maximal longitudinal extent in any direction, the maximum mediolateral extent, the non-collapsing zone, helps select the circle diameter. The circle can then be matched to the unaffected articular margins. Most radiology departments are now equipped with picture archiving and communication systems (PACS), which have integrated software for circle drawing. We used software-based superimposition of the circle to reduce the effort required in the manual drawing of the circle.

The available evidence highlights the importance of the assessment of necrotic lesions for the prediction of future collapse. The combined kerboul angles in AP and lateral views were classically described to measure the extent of osteonecrosis [12]. The use of these angles in coronal and sagittal MRI cuts has been proposed for a more accurate assessment of the necrotic lesion. The higher sum of angles in the two cross-sections is associated with a higher risk of collapse in the future [13]. Besides this, a volumetric assessment through MRI can be used to understand the necrotic lesion’s three-dimensional orientation [14]. However, these methods determine the size of the lesion at one point, and the risk of collapse progression cannot be ascertained purely based on the extent of the necrotic lesion. Currently, there is a lack of quantitative methods for monitoring collapse progression. The MRI scan is an excellent investigation for necrotic lesion assessment. Still, it is difficult to predict how frequently it should be ordered and whether it would be a cost-effective option. Lee and Steinberg [15] rightly pointed out that assessment methods have become more subjective and less quantitative in recent literature. The angular extent measurements discussed above can help measure the maximal extent of the ONFH but can’t tell whether there is collapse progression of the femoral head.

Currently, there is a lack of standardized objective assessment tools for clinical follow-up collapse detection and progression apart from the MRI-based assessment. Although one can measure the femoral head’s deficiency in digitally saved radiographs, the issues due to different radiographic magnifications and lack of scaled digital films can pose difficulty maintaining reliable follow-up. Our method solves these problems by:

1) Using standard AP radiographs for assessment: Standard AP radiographs project the superoinferior extent of the femoral head. The anterosuperior femoral head region is mostly affected in ONFH. The superior part is the direct weight-bearing zone and is thus at risk of collapse. Therefore, AP radiographs can predict the deviation of the femoral head from a round contour. The lateral radiographs, on the other hand, project the anteroposterior extent of the femoral head. The concerns in lateral radiographs are related to difficulties obtaining a true lateral radiograph with similar femoral head projection every time. In addition, ONFH patients often have rotation restrictions that can further limit standardized lateral radiographs’ applicability.

2) Percentage measurements over metric measurement: We quantified the deficient femoral head based on relation to the femoral head diameter. The deficiency was measured as pW, which measured the affected zone as the superimposed circle’s diameter fraction. Thus, the diameter always remained 100% for all radiographs, and the defect zone was expressed as a part of this percentage. This technique can thus be applied to different AP radiographs with variable magnification since the metric values of diameter are not required.

3) Cost-effectiveness: The radiographs are the most readily available radiological investigations and can be used for frequent follow-up. Unlike MRI, radiographic analysis is better feasible when frequent follow-up is required.

Based on the above-specified points, it would be preferable to quantify the ONFH lesion according to size, which can be performed using Kerboul angles, and based on the deficiency in femoral head contour, which can be assessed using the described concentric circle technique.

The current analysis has some limitations. First, the technique helps assess collapse in the superior weight-bearing part of the femoral head, which is best projected in AP radiographs. However, the monitoring of collapse in anterior or posterior articular surfaces can’t be done through this technique. Second, the technique is useful only in pre-arthritic ONFH. Once arthritis has developed, the marginal osteophytes deform the spherical contour of the ONFH unaffected segment. Third, the technique can only be used for monitoring the surface deformation in ONFH. It cannot predict the size of the ONFH lesion. The data from other assessment methods like Kerboul angles can thus play a supplementary role. Fourth, the measurements and their inferences are based on small sample size. Further research on a larger population would be required to strengthen the observations of this technique. Lastly, it may take some time for the surgeons to be familiar with using the described technique as it requires an objective assessment over the conventional non-quantitative methods of radiographic assessment. Nevertheless, the technique fits in well as a reliable method to objectively assess small alterations in femoral head contour in ONFH, which might get missed in conventional assessment. The high interobserver and intraobserver reliability among orthopedic surgeons with different expertise levels suggest an easy learning curve of this technique.

## Conclusions

The described concentric circle technique can act as an objective assessment tool for monitoring subtle alterations in the sphericity of the femoral head and can be helpful for the radiographic follow-up of the ONFH patients. In addition, the technique is simple, reliable, and is not affected by radiographic magnification-related issues. Therefore, we recommend using this technique in combination with standard quantitative radiographic assessment of ONFH.
